# Effects of Augmented Reality Game-Based Cognitive–Motor Training on Restricted and Repetitive Behaviors and Executive Function in Patients with Autism Spectrum Disorder

**DOI:** 10.3390/healthcare10101981

**Published:** 2022-10-09

**Authors:** Daekook M. Nekar, Dong-Yeop Lee, Ji-Heon Hong, Jin-Seop Kim, Seong-Gil Kim, Yong-Gon Seo, Jae-Ho Yu

**Affiliations:** 1 Department of Physical Therapy, Sunmoon University, Asan 31460, Korea; 2 Division of Sports Medicine, Department of Orthopedic Surgery, Samsung Medical Center, Seoul 06351, Korea

**Keywords:** autism spectrum disorder, augmented reality, behavior, executive function, exergame, reaction time

## Abstract

Restricted and repetitive behaviors (RRBs) and executive dysfunction are widely acknowledged as core features and hallmarks in patients with autism spectrum disorder (ASD). This study aimed to investigate the effects of augmented reality (AR) using motivational games with cognitive–motor exercises on RRBs, executive function (EF), attention, and reaction time in patients with ASD. Twenty-four patients (range from 6 to 18 years) diagnosed with ASD were recruited from local social welfare centers and randomly allocated to the AR game-based cognitive–motor training group (study group) or the conventional cognitive training group (control group). Both groups completed 30 min training sessions, twice a week for four weeks. Outcome measures were conducted before and after the intervention. As a result, improvements were observed in all the subscales of the RRBs in the study group except for self-injurious and ritualistic behavior. Significant improvements were observed in EF and reaction time in the study group, which was significantly higher compared to the control group. With the present findings, we can suggest that cognitive–motor training using AR game-based content generates positive effects on improving executive function reaction time and accuracy of responses and has a limited effect on RRBs in patients with ASD. This can be proposed as a complementary intervention associated with individualized daily management.

## 1. Introduction

Autism spectrum disorders (ASD) are a range of complex heterogeneous neurological and developmental disorders identified mainly by deficits in social interaction, delayed or restricted communication, and repetitive and limited patterns of behavior and interests [1]. In the U.S., the prevalence of ASD is approximately 1% in children and adults [2]. The prevalence of ASD in Korea is estimated to be 2.64%, which is more than double that in other countries [3].

Individuals with ASD present with at least one symptom from a set of restricted, repetitive, and stereotyped patterns of behavior. A previous study reported that disorders in selective attention may cause restricted repetitive behaviors (RRBs) [4]. The presence of RRBs in individuals with ASD is due to the abnormal development and functioning of the frontal lobe, which results in executive dysfunction [5,6]. Executive function (EF) is the term used to describe the cognitive process that allows for planning, initiating, and managing tasks and continuing to do so in challenging situations [7]. It includes planning, reasoning ability, cognitive flexibility, cognitive inhibition, working memory, and problem solving [8]. Executive dysfunction, increased reaction time, and disordered selective attention in patients with autism have a major influence on individuals’ general conditions and are a cause of RRBs [6]. Thus, interventions to improve RRBs, EF, selective attention, and reaction time in people with autism may help improve activities of daily life and quality of life.

According to a systematic review, physical exercises, such as hydrotherapy, walking, jogging, ball playing, and aerobic exercises, are beneficial interventions for repetitive behaviors in children with autism [9,10]. Moreover, physical exercise has been reported to have a positive effect on cognitive function, especially EF and attention, in normally developing children as well as in children with autism [11,12]. However, reduced motivation and lack of interest are generally major barriers and limitations for children with autism to participate in normal physical activities. Thus, an individual training program with consistent and adequate feedback for motivation might be a beneficial option for increasing motivation and self-participation in physical activity and sports programs for patients with ASD.

Augmented reality (AR) and virtual reality (VR) have been widely used by healthcare professionals for rehabilitation. VR is a completely immersive technology with a virtual environment that generally requires headsets or accessory devices. However, AR is an interactive platform that superimposes virtual objects in a real environment, without requiring additional devices and is easy to access through different platforms (e.g., computers and smartphones). AR technology may be adequate for training patients with developmental disabilities such as ASD.

Computerized cognitive training is receiving attention as a widely accessible intervention, with various types of devices designed to improve cognitive ability in patients in clinical centers. A tablet version of the Korean computer-based cognitive rehabilitation program (CoTras) was proposed by a team of healthcare professionals for young children and individuals with severe cognitive impairment. The programs were developed with built-in essential and fundamental components of cognitive functions, such as EF, memory, attention, and visual and auditory perception. Previous studies have shown that using CoTras for training improves cognitive function in children with developmental disabilities [13] and can be used as an effective training program to improve cognitive function in adults with dementia [14]. Additionally, it has been proven effective in improving memory in healthy elderly people [15] and cognitive function in patients with impaired cognition, such as patients with cerebrovascular accidents [16,17] and traumatic brain injury [18]. CoTras is widely used in geriatric and pediatric centers and hospitals in Korea for cognitive rehabilitation. Nevertheless, no study has compared the effects of CoTras with those of AR game-based cognitive–motor training on RRBs, EF, attention, and reaction in patients with ASD.

Therefore, the purpose of this study was to investigate the effects of combined cognitive–motor training using AR game content and compare them with those of the tablet version of the computerized CoTras on RRBs, EF, and reaction time in patients with autism.

## 2. Materials and Methods

### 2.1. Study Design

This study used a randomized control trial pretest–posttest design. In accordance with the study protocol, participants were randomly and equally divided into two groups: AR game-based cognitive–motor training group (study group) and tablet-based cognitive training group (control group). Measurements were performed by a trained pediatric physiotherapist before and after the intervention.

### 2.2. Ethics Statement and Trial Registration

The study procedure was conducted in accordance with the principles of the Declaration of Helsinki, approved by the Institutional Review Board of Sunmoon University (SM-202112-072-2), and registered at the Clinical Research Information Service in Korea (CRIS No: KCT0007135). The experiments were conducted at the Department of Physical Therapy at Sunmoon University, Korea. Since the participants were under the age of 18 years, written informed consent to participate in the study was obtained from their parents or legal guardians. Prior to being involved in the experimental procedure, participants and parents/legal guardians were fully informed about the purpose and procedure of the study and were given the opportunity to ask any questions about the study. The consent forms and procedures were approved by the Sunmoon University Research Ethics Committee.

### 2.3. Study Participants

Participants were 24 children and adolescents diagnosed with ASD aged between 6 and 18 years who voluntarily participated in the study with their parents/legal guardians’ written consent. They were recruited from social welfare centers located in Cheonan and Asan City, South Korea.

The inclusion criteria were diagnosis of ASD; ability to see, hear, and understand basic instructions; and ability to read and understand Korean (the main language used on the AR device). The exclusion criteria were genetic conditions (i.e., fragile X syndrome), severe behavioral problems or sensory impairments that potentially limit participants from seeing or hearing cues provided by the AR device, and inability or unwillingness to follow the instructor’s directives. The demographic characteristics of the participants are presented in Table 1.

### 2.4. Randomization and Blinding

Participants were randomly allocated to two groups: the study group (AR game-based cognitive–motor training) and the control group (tablet-based cognitive training). The allocation was conducted by an independent investigator prior to the beginning of the study by producing 24 random numbers. Those with even numbers were assigned to the study group and those with odd numbers were assigned to the control group in a 1:1 ratio. The allocation sequence was concealed using a sealed opaque envelope. Participants did not receive any explanation about how the different groups would perform the training in order to be blind to the type of intervention. All interventions were conducted separately according to the group allocation.

### 2.5. Experiment Procedures

**Figure 1:**
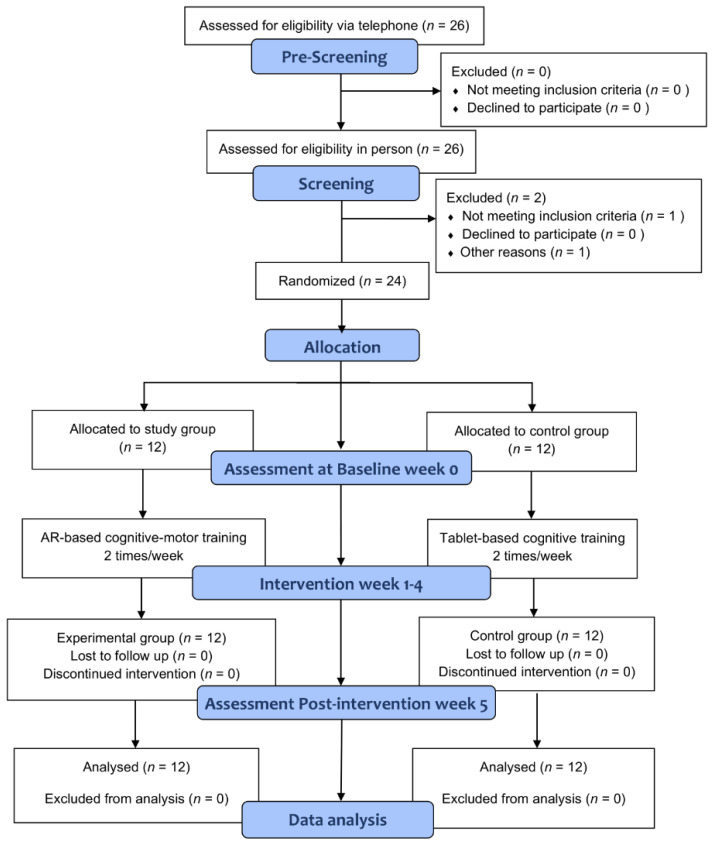
Study flow diagram.

During pre-screening via telephone, 26 participants were interested in participating in the study. However, two participants were excluded after the face-to-face screening. One participant was excluded for personal reasons related to the COVID-19 pandemic and the other presented with severe intellectual and cognitive problems and would, therefore, be unable to follow the instructions. Finally, only 24 participants were included in this study. We could not reach more participants through the social welfare center, parents, and caregivers’ associations. All participants as well as parents/legal guardians of the children involved in this study received a detailed explanation regarding the purpose of the study. Participants were then randomly allocated to either the study or control group and a pre-intervention test consisting of repetitive behavior, EF task accuracy, and reaction time was conducted. After measuring the outcome variables in both groups, the intervention program was performed, followed by a post-intervention test with outcome variables measured again. The intervention program for both groups was conducted twice a week with two sets of 15 min sessions, for a total of 4 weeks. Figure 1 shows a flow diagram of the study procedure.

#### 2.5.1. Study Group

**Figure 2:**
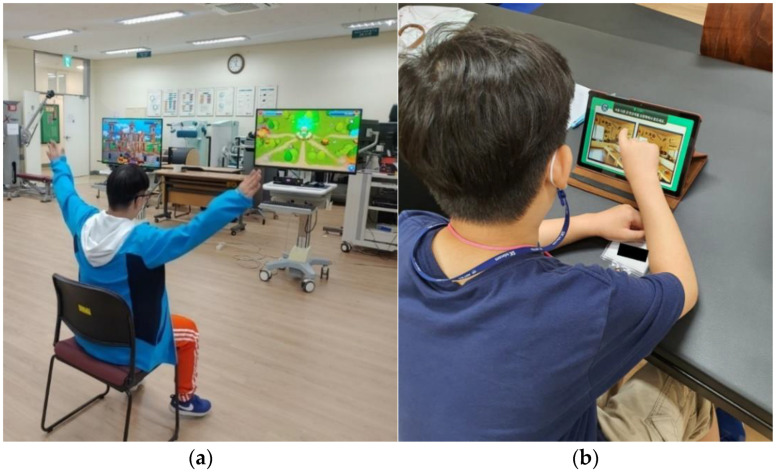
(**a**) AR game-based cognitive–motor training session, (**b**) CoTras-based training session.

Participants allocated to the study group completed two sets of 15 min sessions of combined cognitive and motor training using the game contents of the UINCARE device (UINCARE-82B, UINCARE Corp., Seoul, Korea). It is a rehabilitation device that uses a motion capture system through a Kinect sensor and contains various rehabilitation training programs with real-time audiovisual feedback (Figure 2a). This program can be used without attaching any special markers or sensors. The exercise program consisted of exercises that targeted gross motor movements of the upper extremity, trunk, and lower extremity, and at the same time resolved cognitive tasks such as attention, memory, calculation, and task planning. Details regarding the different games included in the present study are presented in (Supplementary File S1). Instructions were provided prior to performing tasks and participants started the intervention at the lowest difficulty level; when a score > 95% was achieved, the level of difficulty was increased gradually in the next session. During the performance of the games, auditory and visual feedback was provided directly on the screen with words such as “well done”, “perfect”, “great”, and “good”, with a female instructor’s prerecorded voice. The game content used in this study included top-down perspective (bird’s-eye view) games, side-scrolling games, first-person perspective, and third-person perspective games to allow participants to have various visual standpoints. The games were developed on three-dimensional graphics (height, width, and depth) and run on a personal computer with Windows 7 or higher while in the present study it was used on a Windows 10 with a monitor of 1920 × 1080 resolution.

#### 2.5.2. Control Group

Participants allocated to the control group completed two sets of 15 min sessions of cognitive training using the CoTras device. CoTras can provide an individualized training program by adjusting the number of training stimuli, complexity, and speed according to the patient’s condition (Figure 2b). In this study, the exercise program included cognitive tasks, such as decision-making, memory, attention, planning, calculation, object color, shape, and size discrimination. Auditory and visual instructions were provided by the device and instructor.

### 2.6. Outcome Measurements

#### 2.6.1. RRBs

To assess RRBs, we used the Repetitive Behaviors Scale—Revised, which is a standard tool for measuring the repetitive behavior of patients with ASD with strong test–retest reliability (ICC = 0.87 for topographies and 0.90 for frequency) [19]. It consists of 44 items divided into six subscales including stereotyped behavior, self-injurious behavior, compulsive behavior, routine behavior, sameness behavior, and restricted behavior. It is rated on a 4-point scale, with higher scores indicating greater severity of the problem.

#### 2.6.2. EF and Reaction Time

In this study, three core EFs (working memory, cognitive flexibility, and cognitive inhibition) and reaction time were measured at baseline and after the intervention.

A computerized version of the digit span forward was used to measure working memory. The digit span forward is reported to have a concurrent validity of r = 0.44 [20] and test–retest reliability of r = 0.71 [21]. Different numbers appeared one after another on the tablet screen at intervals of 500–1500 ms and then disappeared. Participants were instructed to remember and repeat the sequences of the numbers in the same order by touching the matching number displayed on the right of the screen. A short explanation was given and three trials were performed to familiarize participants with the test and ensure that all participants understood the procedure. After three correct answers, the number of digits increased by one in the next trial and the test ended when participants gave three consecutive incorrect answers. The sum of correct responses was used as the digit span forward score.

A computerized version of the Wisconsin Card Sorting Test (WCST) was used to assess cognitive flexibility. The WCST has split-half reliability that ranges between 0.90 and 0.95 and is considered one of the standard measurement tools for cognitive flexibility [22]. The test consists of four stimulus cards with three dimensions (color, shape, and number). Among 64 cards varying along the same dimensions, participants were asked to match the cards in the deck with one of the fourth stimulus cards. The sum of correct answers and mean reaction time were recorded.

The computerized version of the Stroop color-word test was used to assess cognitive inhibition. Regarding the main scores of Word, Color, and Color–Word, research has reported good reliability with r > 0.80 [23]. The screen displayed the word “red”, “green”, “yellow”, or “blue” on a randomly changing colored background in either red, green, yellow, or blue text. The trials were divided into congruent and incongruent. In congruent trials, the word and text color in which it was displayed matched. Incongruent trials consisted of those in which the word and text color did not match. The words appeared in random order at intervals of 500 and 1500 ms. The sum of correct responses and the mean reaction time were recorded.

### 2.7. Data Analysis

SPSS software version 26.0 for Windows (SPSS Inc., Chicago, IL, USA) was used for statistical analysis and the data are presented as mean ± standard deviation. Descriptive statistics were used to analyze the general characteristics of participants. A 2 × 2 mixed ANOVA with one within-subject factor (time: pretest and posttest) and one between-subject factor (group: study and control group) was conducted for the effects of the intervention and their interaction. The Shapiro–Wilk test was used to analyze the normality of the demographic data and outcome variables. It showed an abnormal distribution of some data; thus, non-parametric tests were conducted for the analysis of the variables. The Mann–Whitney U-test was used to compare the mean difference in the baseline data between the study and control groups. The Wilcoxon signed-rank test was used to analyze changes over time within groups and the Mann–Whitney U-test was used to compare differences in values between the groups. The significance level for the tests was set at *p* < 0.05.

## 3. Results

Twenty-six patients were prescreened via telephone and two were excluded after the face-to-face screening. Thus, 24 were included in this study. There were no dropouts during the experiment. The demographic characteristics of participants in both groups are shown in Table 1. There were no statistically significant differences in age, height, weight, or mental status between the intervention and control groups at baseline for all participants.

### 3.1. RRBs

The results of all RRBs subscales are displayed in Table 2. First, the ANOVA result showed significant differences between the pretest and posttest scores of all the subscales with *p* < 0.05. Furthermore, the within-group comparison of the main effect showed a statistically significant improvement in stereotypical behavior, compulsive behavior, sameness behavior, and restricted behavior in the study group (*p* = 0.029, *p* = 0.021, *p* = 0.046, and *p* = 0.022, respectively). In the control group, compulsive behavior and restricted behavior were significantly improved (*p* = 0.035 and *p* = 0.046, respectively).

Second, the ANOVA result showed no significant interaction of time × group in all RRBs subscales, while no significant difference was observed between the two groups in any of the restricted or repetitive subscales (*p* > 0.05).

### 3.2. EF and Reaction Time

Table 3 shows the results of the working memory score, reaction time, and accuracy percentage for cognitive flexibility and cognitive inhibition. The ANOVA analysis showed significant differences in the within-subject factor of pretest and posttest in memory and cognitive flexibility and cognitive reaction time as well as the accuracy of response (*p* < 0.05). Additionally, the within-group comparison showed a significant improvement in the working memory score (*p* = 0.032), cognitive flexibility reaction time (*p* = 0.002), cognitive flexibility response accuracy (*p* = 0.005), cognitive inhibition reaction time (*p* = 0.016), and response accuracy (*p* = 0.002) in the study group. In the control group, the working memory score, cognitive flexibility reaction time, and response accuracy showed significant improvements (*p* = 0.048, *p* = 0.041, and *p* = 0.023, respectively).

The ANOVA showed significant differences in the time × group, indicating interactions between the groups. During the between-group comparison, significant differences were observed in cognitive flexibility, cognitive inhibition reaction time, and response accuracy. The study group showed greater improvement than the control group.

## 4. Discussion

This study aimed to investigate the effects of AR game-based cognitive–motor training on different components of RRBs, EF, and reaction time in patients with ASD. The main findings of this study are the significant improvements in all subscales of RRBs after the intervention, except for self-injuries and ritualistic behaviors. All three core EF accuracies and reaction times also improved after the performance of the games in the study group.

The AR game-based cognitive–motor exercises in the study group demonstrated a significant decrease in purposeless movements or actions. These results can be explained by the effects of the application of motor tasks with simultaneous cognitive exercise during training. A previous study [24] suggests that the decrease in stereotypic behaviors in individuals with ASD occurs with an increase in physical exertion, which is the basis of the theory of fatigue after exercise-decreasing behaviors. A possible explanation is that the stimulation produced during the motor task may be similar to self-stimulatory behavior. The similarity of the stimulation provided by the AR game content may be a factor that reduces the experience of stereotypic behavior. Moreover, we suggest that the simultaneous performance of cognitive tasks and real-time feedback provided along with the AR game-based cognitive–motor training enhanced the motivational level and self-stimulation. Thus, motivational theory can be considered another major factor that compensates for involuntary self-stimulation and supports our results. Some of the common stereotypic behaviors observed in our participants were body rocking, sudden running, swaying, hand/finger flapping, waving or shaking hands, clapping, turning in circles, spinning, and twirling objects. We remarked that these behaviors generally occur during frustration, anxiety, or stressful stimuli. Thus, individuals with ASD may perform these behaviors as automatic reinforcement or sensory input because they produce internally favorable and enjoyable stimuli. The explanation for the decrease in stereotypic behavior in our study is supported by a previous study that found that reinforcement of object manipulation decreased stereotypic behaviors in children with ASD [25].

Although no significant improvement was observed in the self-injurious behavior subscale, a decrease was observed in both groups. Self-injurious behaviors, similar to stereotypic behaviors, are a form of self-stimulation, but on an extreme level, resulting in injury to oneself. The result of the self-injurious behavior obtained in our study can be attributed to the theory that self-injurious behaviors result from maladaptive strategies used to manage emotions (anxiety and stress) and physiological arousal. This mechanism includes the upregulation and downregulation of arousal states [26]. Individuals with high physiological arousal may exhibit self-injurious behaviors to downregulate the arousal level, whereas those with low physiological arousal may exhibit such behaviors to create stimulation and elevate the arousal level [27,28]. In our study, we estimated that participants with high arousal levels could not reduce the exhibition of self-injurious behaviors since the combination of cognitive–motor exercises may have a high demand on brain function, especially in the frontal cortex. Moreover, it is necessary to note that participants included in the present study presented a relatively lower mean of self-injurious behavior during the overall experiment.

Regarding the second category of RRBs, which is difficulty in changing compulsive behaviors, routines, rituals, and restricted interests, significant improvements were observed in all subscales in the study group after the intervention, with the exception of ritualistic behavior. According to previous theories, this result can be explained by the etiological mechanism of these behaviors and effects of cognitive–motor dual-task training on certain aspects of brain functioning. Neurobiological studies suggest the presence of abnormalities in communication between the cortex, striatum, and thalamus (cortico-striatal-thalamo-cortical pathway) in individuals with obsessive and compulsive behaviors, such as obsessive-compulsive disorder and ASD [29]. Thus, the problem of communication between the three core brain structures may be the reason for the breach and sticks in the repetition of the same loops of thought and behavior [30]. In addition, the dysregulation of neurotransmitters, such as serotonin (regulation of memory, behavior, learning, mood, and sensory perception), dopamine (attention and focus), and gamma-aminobutyric acid (GABA; regulation of brain activity), is involved in the rigidity and repetition of thought and behaviors [30]. Holschneider et al. [31] found that exercise can cause functional and morphological modifications in the brain. Based on this assumption, we can suggest that decreases in compulsive, sameness, and restricted behaviors may be related to the reorganization of the functional brain and synchronization of the cortico-striatal-thalamo-cortical pathway, as well as the regulation of serotonin, dopamine, and GABA. However, the specific effect of dual tasks on the regulation of neurotransmitters and brain function remains unclear. Henceforth, questions concerning the implications of the cortico-striatal-thalamo-cortical pathway and the above neurotransmitters for the presence of RRBs are still disputable. We can add the potential effect of similarity in stimulation and motivational theory to reductions in these behaviors.

In the present study, we found that AR game-based cognitive–motor training improved all core EF subtests, including working memory, cognitive flexibility, and cognitive inhibition. More importantly, we presume that these improvements in EF are related to improvements in the RRB subscales. The current findings are consistent with those of previous studies [11,32,33], in which AR/VR game-based training was shown to improve core EF in children with ASD. The neurophysiological explanation of why EF may be improved by exercise is still not fully understood. However, these hypotheses involve prompt improvement in cerebral blood flow, delivery of oxygen and nutrients, and removal of byproducts [34]. Cognitive flexibility, cognitive inhibition, and reaction time depend on the activity of the prefrontal cortex and its associated regions, such as the cingulate cortex [35]. Furthermore, working memory is closely related to the activity of the frontal and parietal networks [36]. We assume that these regions of the brain are sufficiently activated during cognitive–motor training using an AR device with motivational game content. Hence, our theory explaining the findings of this study is that cognitive–motor training has a higher demand to challenge the cognitive process capable of inducing changes in cognitive functioning.

Only cognitive flexibility, cognitive inhibition reaction time, and response accuracy showed statistically significant differences between the groups. This result can be explained by the different nature of intervention methods. First, the contents included in the AR game-based cognitive–motor training allowed for not only the processing of cognitive functions, such as attention and memory, but also the ability to balance and switch simultaneously between the demands of the motor tasks. In other words, participants who practiced cognitive training alone had less demand for simultaneously shifting between tasks of different natures and were more fixated on the single ongoing task.

It is important to note that in the present study, the ritualistic behavior subscale showed a consistent improvement after AR game-based cognitive–motor training, but without a statistically significant difference. This result can be explained by the fact that the behaviors included in the ritualistic behavior subscale are all experiments during activities of daily living, such as a strong preference for a particular food, insistence on the presence of another person prior to sleep, and performing certain routes. These behaviors are strongly associated with social interaction and anxiety and are influenced by the surrounding environment.

This study has several limitations that need to be acknowledged. First, the sample size of participants involved was very small with a total of *n* = 24 and 12 participants per group, which limits the generalization of the results and the analysis of the statistical effect size. Second, we could not assess the educational and intellectual level of the participants. As this study included cognitive tasks, such as calculation and memory tasks, the educational and intellectual levels of participants might have affected the outcomes. Another limitation is the short duration of the training program, which, at some point, may influence the magnitude of the training effect on behavioral or cognitive outcomes. It would be interesting to repetitively (weekly) investigate in future research the effects of cognitive–motor training on behavioral and cognitive outcomes, along with its long-term impact on the participant’s interest, since we know that individuals with ASD present restricted interest. In addition to these limitations, it is important to mention that no harmful effects were observed in any participant during or after the program.

## 5. Conclusions

The present study investigated the effects of AR game-based cognitive–motor training on RRBs, EF, and reaction time in patients with ASD. The findings revealed positive effects of cognitive–motor training using games on AR devices for improving stereotypic behaviors, compulsiveness, sameness, and restricted behaviors, as well as working memory, cognitive flexibility, cognitive inhibition reaction time, and attention, after 4 weeks of training in patients with ASD. Further research is needed to provide additional evidence concerning this mechanism and to evaluate the long-term effect of AR on individuals with ASD, along with the capacity to maintain interest in this form of training.

## Figures and Tables

**Table 1:** Participants’ demographic characteristics.

Variables	Study Group (*n* = 12)	Control Group (*n* = 12)	*p*
Male/Female (%)	10/2 (83/17)	12/0 (100/0)	-
Age (years)	14.42 ± 5.14	14.17 ± 5.09	0.977
Height (cm)	155.42 ± 11.64	153.42 ± 11.75	0.525
Weight (kg)	53.42 ± 9.52	53.33 ± 11.33	0.840
AMSE (points)	3.33 ± 1.30	3.58 ± 1.31	0.651

Mean ± standard deviation, AMSE: autism mental status exam.

**Table 2:** The results of restricted and repetitive behaviors within and between-group comparison.

Variables	Study Group (*n* = 12)		Control Group (*n* = 12)			Mixed 2 × 2 ANOVA	
	Pre-Test	Post-Test	Pre-Test	Post-Test	*p*	Time F-Value	Time × Group F-Value
Stereotypic behavior (points)	5.50 ± 1.00	4.75 ± 1.05 *	5.92 ± 1.24	5.50 ± 1.67	0.215	11.846 **	0.967
Self-injury behavior (points)	1.75 ± 0.75	1.25 ± 0.86	1.83 ± 0.83	1.33 ± 1.07	0.778	7.333 *	0.000 ***
Compulsive behavior (points)	3.50 ± 1.00	2.83 ± 1.33 *	4.00 ± 1.34	3.42 ± 1.50 *	0.749	15.184 **	0.067
Ritualistic behavior (points)	7.00 ± 1.12	6.33 ± 1.72	6.83 ± 1.11	6.25 ± 1.48	0.833	8.756 **	0.039 *
Sameness behavior (points)	8.50 ± 0.90	7.67 ± 1.15 *	8.92 ± 1.37	8.58 ± 1.73	0.289	8.045 *	1.478
Restricted behavior (points)	3.50 ± 0.90	2.50 ± 1.44 *	3.75 ± 1.21	3.08 ± 1.50 *	0.426	13.750 **	0.550

Data are mean ± standard deviation. Means a significant difference within group (Wilcoxon-signed-rank test). *p*-value is described between the groups (Mann–Whitney U-test and mixed 2 × 2 ANOVA), *p* < 0.05 *, *p* < 0.01 **, *p* < 0.001 ***.

**Table 3:** The results of executive function and reaction time within and between-group comparison.

Variables		Study Group (*n* = 12)		Control Group (*n* = 12)			Mixed 2 × 2 ANOVA	
		Pre-Test	Post-Test	Pre-Test	Post-Test	*p*	Time F-Value	Time × Group F-Value
Working memory (points)		13.92 ± 1.50	13.92 ± 1.50 *	14.08 ± 1.83	15.17 ± 1.19*	0.977	11.407 **	0.016
Cognitive Flexibility	RT (milliseconds)	1102.08 ± 5.46	1092.25 ± 5.15 **	1102.00 ± 5.20	1098.58 ± 5.12*	0.016	30.630 ***	7.184 *
	AR (points)	79.08 ± 3.02	84.83 ± 2.40 **	79.25 ± 4.11	81.92 ± 2.42 *	0.043	12.950 **	6.349 *
Cognitive Inhibition	RT (milliseconds)	1104.92 ± 8.17	1102.17 ± 7.34 *	1104.83 ± 5.13	1104.25 ± 5.17	0.045	12.308 **	5.200 *
	AR (points)	76.08 ± 2.06	84.33 ± 2.10 **	76.33 ± 3.79	81.08 ± 2.42 *	0.042	14.503 **	4.351 *

Mean ± standard deviation,. RT: reaction time, AR: Accuracy of responses. Means a significant difference within group (Wilcoxon-signed-rank test). *p*-value is described between the groups (Mann–Whitney U-test and mixed 2 × 2 ANOVA). *p* < 0.05 *, *p* < 0.01 **, *p* < 0.001 ***.

## Data Availability

The data used to support the findings of this study are available from the corresponding author upon reasonable request.

## Supplementary Materials

The following supporting information can be downloaded at: https://www.mdpi.com/article/10.3390/healthcare10101981/s1, Supplementary File S1: Selected game contents.

## References

[1] American Psychiatric Association (2013). Diagnostic and Statistical Manual of Mental Disorders (DSM-V).

[2] Centers for Disease Control and Prevention (2009). Prevalence of Autism Spectrum Disorders-Autism and Developmental Disabilities Monitoring Network, United States, 2006.

[3] Kim Y.S., Leventhal B.L., Koh Y.J., Fombonne E., Laska E., Lim E.C., Cheon K.A., Kim S.J., Kim Y.K., Lee H. (2011). Prevalence of autism spectrum disorders in a total population sample. Am. J. Psychiatry.

[4] Courchesne E., Allen G. (1997). Prediction and preparation, fundamental functions of the cerebellum. Learn. Mem..

[5] Lopez B.R., Lincoln A.J., Ozonoff S., Lai Z. (2005). Examining the relationship between executive functions and restricted, repetitive symptoms of autistic disorder. J. Autism Dev. Disord..

[6] Turner M. (1999). Annotation: Repetitive behaviour in autism: A review of psychological research. J. Child Psychol. Psychiatry Allied Discip..

[7] Roselló B., Berenguer C., Navío P., Baixauli I., Miranda A. (2017). Executive functioning, social cognition, pragmatics, and social interaction in attention deficit hyperactivity disorder and autism spectrum disorder. Curr. Dev. Disord. Rep..

[8] Hill E.L. (2004). Evaluating the theory of executive dysfunction in autism. Dev. Rev..

[9] Petrus C., Adamson S.R., Block L., Einarson S.J., Sharifnejad M., Harris S.R. (2008). Effects of exercise interventions on stereotypic behaviours in children with autism spectrum disorder. Physiother. Can..

[10] Lang R., Koegel L.K., Ashbaugh K., Regester A., Ence W., Smith W. (2010). Physical exercise and individuals with autism spectrum disorders: A systematic review. Res. Autism Spectr. Disord..

[11] Ji C., Yang J., Lin L., Chen S. (2022). Executive Function Improvement for Children with Autism Spectrum Disorder: A Comparative Study between Virtual Training and Physical Exercise Methods. Children.

[12] Tse A.N., Anderson D.A., Liu V.E., Tsui S.H. (2021). Improving Executive Function of Children with Autism Spectrum Disorder through Cycling Skill Acquisition. Med. Sci. Sport Exer..

[13] Park J.H., Park J.H. (2015). A randomized controlled trial of the computer-based cognitive rehabilitation program for children (CoTras-C) to examine cognitive function and visual perception in children with developmental disabilities. J. Phys. Ther. Sci..

[14] Kim S.Y., Choi Y.I. (2019). Effects of a computerized cognitive training on cognitive, depression, life satisfaction and activity of daily living in older adults with mild dementia. J. Korea Acad.-Ind. Coop. Soc..

[15] Lee J.S., Kim S.W. (2018). Effects of Korean Computer-Based Cognitive Rehabilitation Program on the Memory in Healthy Elderly. J. Int. Acad. Phys. Ther. Res..

[16] Jang C., Bae W.S. (2021). The Effect of Computerized Cognitive Program on Cognitive Function and Activities of Daily Living of Stroke Patients. J. Korean Soc. Integr. Med..

[17] Kim M., Park J., Lee N. (2020). The effect of the computer-based cognitive rehabilitation program (CoTras) on the cognitive function and daily living activities of elderly stroke patients. J. Korean Soc. Integr. Med..

[18] Han S.H., Jo E.J., Noh D.H., Kam K.Y. (2015). Effects of Korean Computer-Based Cognitive Rehabilitation Program (CoTras) on Frontal-Executive Functions in Patients with Traumatic Brain Injury. J. Korea Acad.-Ind. Coop. Soc..

[19] Wolff J.J., Boyd B.A., Elison J.T. (2016). A quantitative measure of restricted and repetitive behaviors for early childhood. J. Neurodev. Disord..

[20] Gualtieri C.T., Johnson L.G. (2006). Reliability and validity of a computerized neurocognitive test battery, CNS Vital Signs. Arch. Clin. Neuropsychol..

[21] Dong Y., Thompson C.L., Tan S.H., Lim L.B., Pang W., Chen C.L. (2013). Test-retest reliability, convergent validity and practice effects of the RBANS in a memory clinic setting: A pilot study. Open J. Med. Psychol..

[22] Kopp B., Lange F., Steinke A. (2021). The reliability of the Wisconsin card sorting test in clinical practice. Assessment.

[23] Homack S., Riccio C.A. (2004). A meta-analysis of the sensitivity and specificity of the Stroop Color and Word Test with children. Arch. Clin. Neuropsychol..

[24] Bachman J.E., Fuqua R.W. (1983). Management of inappropriate behaviors of trainable mentally impaired students using antecedent exercise. J. Appl. Behav. Anal..

[25] Rapp J.T., Vollmer T.R., St Peter C., Dozier C.L., Cotnoir N.M. (2004). Analysis of response allocation in individuals with multiple forms of stereotyped behavior. J. Appl. Behav. Anal..

[26] Nock M.K., Prinstein M.J. (2005). Contextual features and behavioral functions of self-mutilation among adolescents. J. Abnorm. Psychol..

[27] Nock M.K., Mendes W.B. (2008). Physiological arousal, distress tolerance, and social problem-solving deficits among adolescent self-injurers. J. Consult. Clin. Psychol..

[28] Selby E.A., Nock M.K., Kranzler A. (2014). How does self-injury feel? Examining automatic positive reinforcement in adolescent self-injurers with experience sampling. Psychiatry Res..

[29] Jacob S., Landeros-Weisenberger A., Leckman J.F. (2009). Autism spectrum and obsessive–compulsive disorders: OC behaviors, phenotypes and genetics. Autism Res..

[30] Minshew N.J., Williams D.L. (2007). The new neurobiology of autism: Cortex, connectivity, and neuronal organization. Arch. Neurol..

[31] Holschneider D.P., Yang J., Guo Y., Maarek J.M. (2007). Reorganization of functional brain maps after exercise training: Importance of cerebellar–thalamic–cortical pathway. Brain Res..

[32] Rafiei Milajerdi H., Sheikh M., Najafabadi M.G., Saghaei B., Naghdi N., Dewey D. (2021). The effects of physical activity and exergaming on motor skills and executive functions in children with autism spectrum disorder. Games Health J..

[33] Anderson-Hanley C., Tureck K., Schneiderman R.L. (2011). Autism and exergaming: Effects on repetitive behaviors and cognition. Psychol. Res. Behav. Manag..

[34] Bremer E., Graham J.D., Heisz J.J., Cairney J. (2020). Effect of acute exercise on prefrontal oxygenation and inhibitory control among male children with autism spectrum disorder: An exploratory study. Front. Behav. Neurosci..

[35] Friedman N.P., Robbins T.W. (2022). The role of prefrontal cortex in cognitive control and executive function. Neuropsychopharmacology.

[36] Eriksson J., Vogel E.K., Lansner A., Bergström F., Nyberg L. (2015). Neurocognitive architecture of working memory. Neuron.

