# Supplementary material for: Effects of Augmented Reality Game-Based Cognitive–Motor Training on Restricted and Repetitive Behaviors and Executive Function in Patients with Autism Spectrum Disorder
Source: Healthcare (Basel). 2022 Oct 9;10(10):1981. doi: 10.3390/healthcare10101981 (PMC9602056; doi:10.3390/healthcare10101981)
Supplement: Supplementary file 1 [file healthcare-10-01981-s001.zip › healthcare-1889412-supplementary.pptx]

## Slide 1
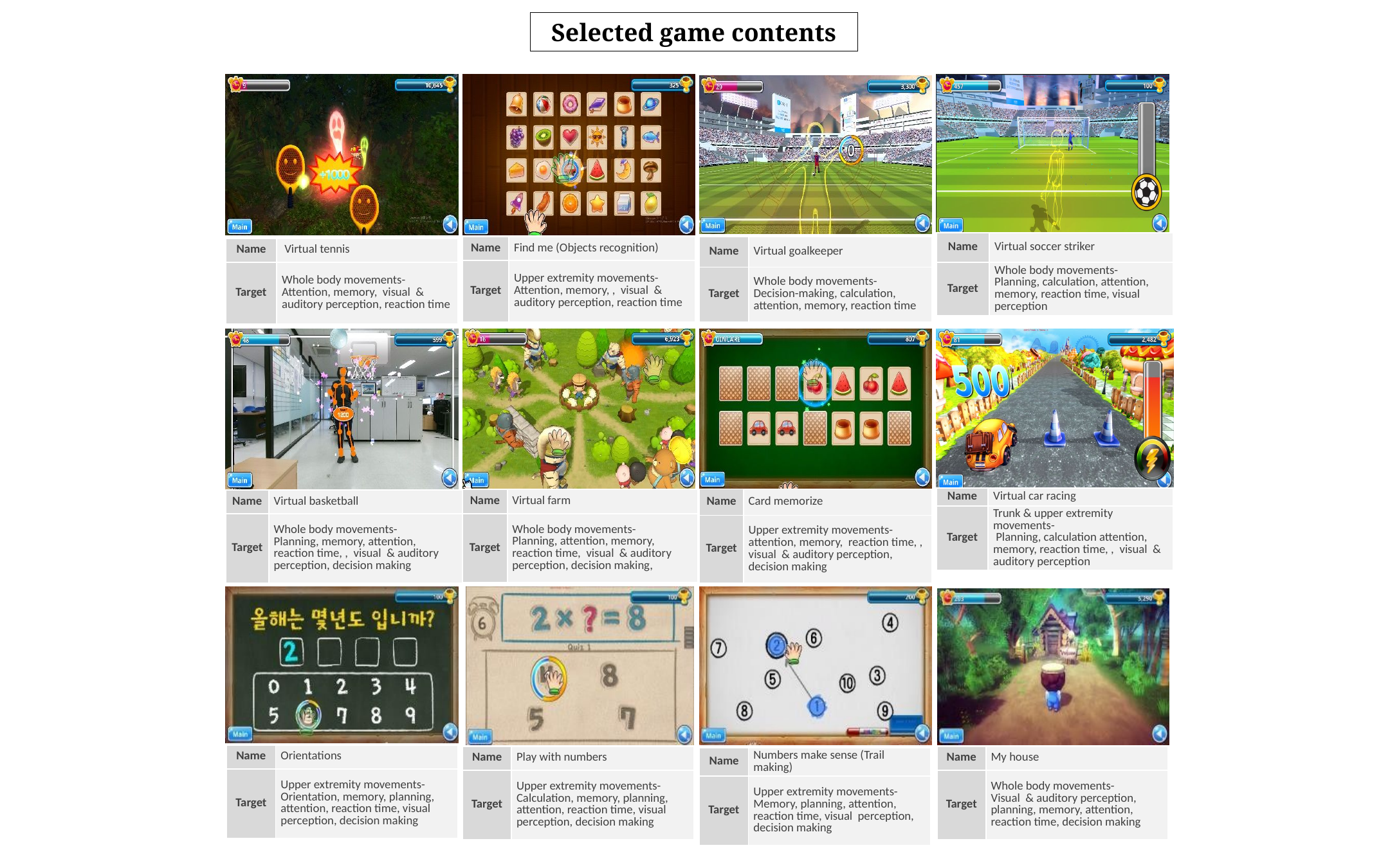

Selected game contents
| Name | Virtual soccer striker |
| --- | --- |
| Target | Whole body movements- Planning, calculation, attention, memory, reaction time, visual perception |
| Name | Virtual goalkeeper |
| --- | --- |
| Target | Whole body movements- Decision-making, calculation, attention, memory, reaction time |
| Name | Find me (Objects recognition) |
| --- | --- |
| Target | Upper extremity movements- Attention, memory, , visual & auditory perception, reaction time |
| Name | Virtual tennis |
| --- | --- |
| Target | Whole body movements-Attention, memory, visual & auditory perception, reaction time |
| Name | Virtual car racing |
| --- | --- |
| Target | Trunk & upper extremity movements- Planning, calculation attention, memory, reaction time, , visual & auditory perception |
| Name | Card memorize |
| --- | --- |
| Target | Upper extremity movements- attention, memory, reaction time, , visual & auditory perception, decision making |
| Name | Virtual farm |
| --- | --- |
| Target | Whole body movements- Planning, attention, memory, reaction time, visual & auditory perception, decision making, |
| Name | Virtual basketball |
| --- | --- |
| Target | Whole body movements- Planning, memory, attention, reaction time, , visual & auditory perception, decision making |
| Name | Orientations |
| --- | --- |
| Target | Upper extremity movements- Orientation, memory, planning, attention, reaction time, visual perception, decision making |
| Name | My house |
| --- | --- |
| Target | Whole body movements- Visual & auditory perception, planning, memory, attention, reaction time, decision making |
| Name | Play with numbers |
| --- | --- |
| Target | Upper extremity movements- Calculation, memory, planning, attention, reaction time, visual perception, decision making |
| Name | Numbers make sense (Trail making) |
| --- | --- |
| Target | Upper extremity movements- Memory, planning, attention, reaction time, visual perception, decision making |
